# Community-based amoxicillin treatment for fast breathing pneumonia in young infants 7–59 days old: a cluster randomised trial in rural Bangladesh, Ethiopia, India and Malawi

**DOI:** 10.1136/bmjgh-2021-006578

**Published:** 2021-08-20

**Authors:** Enhanced Management of Pneumonia in Community (EMPIC) Study, Yasir B Nisar

**Affiliations:** Department of Maternal, Newborn, Child and Adolescent Health and Ageing, WHO, Geneve, Switzerland

**Keywords:** child health, paediatrics, pneumonia, cluster randomized trial

## Abstract

**Introduction:**

Young infants 7–59 days old with fast breathing pneumonia presented to a primary level health facility receive a 7-day course of amoxicillin as per the WHO guideline. However, community-level health workers (CLHW) are not allowed to treat these infants. This trial evaluated the community level treatment of non-hypoxaemic young infants with fast breathing pneumonia by CLHWs.

**Methods:**

This cluster-randomised, open-label, non-inferiority trial was conducted in rural areas of Bangladesh, Ethiopia, India and Malawi. We randomly allocated clusters (first-level health facility) 1:1, stratified by the population size, to an intervention group (enhanced community case management) or control group (standard community case management). Infants aged 7–59 days with a respiratory rate of ≥60 breaths/min and oxygen saturation (SpO_2_) ≥90% were enrolled. In the intervention clusters, these infants were treated with a 7-day course of oral amoxicillin (according to WHO weight bands) and were regularly followed up by CLHWs. In the control clusters, CLHWs continued the standard management (assess and refer after pre-referral antibiotic dose) and followed up according to the national programme guideline. The primary outcome of treatment failure was assessed in both groups by independent outcome assessors on days 6 and 14 after enrolment. Secondary outcomes (accuracy and impact of pulse oximetry) were also assessed.

**Results:**

Between September 2016 and December 2018, we enrolled 2334 infants (1168 in intervention and 1166 in control clusters) from 208 clusters (104 intervention and 104 control). Of 2334, 22 infants with fast breathing were excluded from analysis, leaving 2312 (1155 in intervention clusters and 1157 in control clusters) for intention-to-treat analysis. The proportion of treatment failure was 5.4% (63/1155) in intervention and 6.3% (73/1157) in the control clusters, including two deaths (0.2%) in each group. The adjusted risk difference for treatment failure between the two groups was −1.0% (95% CI −3.0% to 1.1%). The secondary outcome showed that CLHWs in the intervention clusters performed all recommended steps of pulse oximetry assessment in 94% (1050/1115) of enrolled patients.

**Conclusions:**

The 7-day amoxicillin treatment for 7–59 days old non-hypoxaemic infants with fast breathing pneumonia by CLHWs was non-inferior to the currently recommended referral strategy.

**Trial registration numbers:**

CTRI/2017/02/007761 and ACTRN12617000857303.

**Key questions**

**What is already known?**

- WHO guideline on managing possible serious bacterial infection in young infants when referral is not feasible recommends outpatient treatment for fast breathing pneumonia in young infants 7–59 days of age with oral amoxicillin by trained health workers at a primary level health facility.
- Several studies have shown that outpatient treatment by the facility level health workers with a 7-day course of oral amoxicillin for young infants with fast breathing is safe and effective in programme settings.
- This WHO guideline recommends that community-level health workers (CLHWs) should assess these infants and, if fast breathing is present, refer them immediately to a referral facility offering them a single pre-referral antibiotic dose.
- However, a high rate of non-compliance to referral advice has been reported in several studies, resulting in low pneumonia treatment coverage.

**Key questions**

**What are the new findings?**

- CLHWs appropriately assessed young infants 7–59 days old with fast breathing and other danger signs in intervention and control clusters. In the intervention clusters, CLHWs performed pulse oximetry to identify hypoxaemic infants.
- CLHWs in the intervention clusters treated non-hypoxaemic infants with fast breathing pneumonia with a 7-day course of oral amoxicillin in the community, whereas in the control clusters, CLHWs referred infants with fast breathing pneumonia to a referral facility as per the standard management.
- The adjusted risk difference in the treatment failure rates between the intervention and the control groups was 1%, which satisfied the prespecified non-inferiority criterion of the intervention to the control clusters.
- CLHWs correctly performed pulse oximetry in 94.2% of cases in the intervention clusters.

**What do the new findings imply?**

- This evidence shows that the current integrated community case management algorithm can be reviewed to further improve pneumonia management in the community setting, resulting in prompt treatment and reduced referrals.
- It has major public health implication for reducing infant mortality in low-income and middle-income countries where pneumonia is a major contributor to child mortality.

## Introduction

Pneumonia causes over 800 000 childhood deaths, and the burden is disproportionately borne by those living in sub-Saharan Africa and South Asia.^1^ To address major childhood illnesses, including pneumonia, WHO and UNICEF developed integrated management of childhood illness (IMCI) for health workers at primary care facility^2^ and integrated community case management (iCCM) protocol for community-level health workers (CLHWs) to manage common childhood illnesses.^3^ In 2015, WHO recommended that young infants with fast breathing be treated on an outpatient basis with oral amoxicillin without referral^4^ and later revised the young infant component of IMCI.^5^ Afterwards, several observational studies from Africa and Asia have shown that outpatient treatment of fast breathing in young infants with oral amoxicillin is safe and effective in programme settings at a primary health facility level.^6–12^ However, this WHO guideline did not extend to iCCM protocol due to paucity of evidence, which recommends young infants with fast breathing be referred to a health facility.^3^ A high rate of non-compliance to referral advice has been reported in several studies from low-income and middle-income countries, particularly in young infants.^13–18^

WHO/UNICEF iCCM was developed to increase access to treatment for pneumonia, malaria and diarrhoea as they are major causes of childhood deaths.^19^ Reduction in pneumonia and child mortality has been demonstrated using CCM by CLHWs when access to health facilities was inadequate,^20 21^ but insufficient evidence was available for under 2 months old infants. A small number of neonates with pneumonia were successfully managed with oral cotrimoxazole at the community level by CLHWs in India for the first time in the early 1990s in an observational study.^22^ Other studies in Bangladesh, India, Nepal, Tanzania and Pakistan demonstrated the efficacy of pneumonia case management at the community level through CLHW in under 5-year-old children, but they did not separately report the data for young infants.^23–28^

A recent systematic review reported five times higher odds of pneumonia-related death among under-five children with hypoxaemia (defined as oxygen saturation (SpO_2_) <90%).^29^ The WHO recommends oxygen for <90% SpO_2_.^30^ A pulse oximeter can non-invasively measure peripheral SpO_2_, and its use at the outpatient level has the potential to identify pneumonia with hypoxaemia for immediate referral for oxygen and injectable antibiotics to a hospital. The IMCI chart booklet recommends pulse oximeter use by a trained healthcare worker to assess hypoxaemia in children 2–59 months of age with cough and/or difficulty breathing at a primary level healthcare facility.^2^ However, the iCCM protocol does not recommend the use of a pulse oximeter by a CLHW.^3^

If this evidence gap could be filled, it will potentially increase access to pneumonia treatment in young infants in low resource, and humanitarian settings where seeking care at referral level health facilities is challenging. Thus, we conducted a trial to evaluate the community level treatment of fast breathing pneumonia in young infants 7–59 days old by CLHWs.

## Methods

### Study design and study participants

We conducted a multicountry cluster-randomised controlled, open-label, non-inferiority trial of the enhanced pneumonia component of iCCM, defined in box 1, versus a standard pneumonia component of iCCM. Clusters were randomised to either a control (standard iCCM) or intervention group (enhanced iCCM). Two African (Ethiopia and Malawi) and two Asian (Bangladesh and India) sites were selected based on the burden of pneumonia disease and a national government-supported functional iCCM programme that allowed CLHWs to treat pneumonia in the community with oral antibiotics.

Box 1 **Definition of community case management (pneumonia component) in intervention and control clusters^33^ and list of danger signs assessed in the study^3^**

**Part A: Community case management (pneumonia component)**

- Intervention clusters—enhanced community case management (pneumonia component):
  - Assess fast breathing and danger signs (as given below) in young infants 7–59 days old.
  - Perform pulse oximetry and refer hypoxaemic young infants or infants with other danger signs (as given below) to a referral facility/hospital.
  - Treat non-hypoxaemic young infants 7–59 days old with fast breathing only (≥60 breaths per min) with a 7-day course of oral amoxicillin.
- Control clusters—standard community case management (pneumonia component):
  - Assess fast breathing and danger signs (as given below) in young infants 7–59 days old.
  - Refer the sick young infant (including fast breathing only) to a referral facility/hospital.

**Part B: Danger signs assessed in the study**

- Not able to feed at all or stopped feeding well.
- Convulsions or fits.
- Movement only when stimulated or no movement at all.
- Severe chest indrawing.
- High body temperature (≥38°C).
- Low body temperature (<35.5°C).
- Local infection* (red or draining umbilicus, skin pustules).
- Yellow soles (jaundice).
- Diarrhoea*.
- Low weight (<2000 g).

*In Bangladesh, as per their national policy, these signs were not considered as danger signs requiring referral.

In this study, young infants aged 7–59 days with fast breathing, defined as the respiratory rate of 60 or more breaths per min, were assessed by trained CLHWs for fast breathing, danger signs given in box 1 (in both intervention and control clusters) and hypoxaemia (by CLHWs in intervention clusters and by study supervisors in control clusters) for enrolment. Non-hypoxaemic infants with fast breathing only, with no danger signs, with informed written consent and previously not enrolled in this study were included and followed for study outcomes.

### Ethics and quality assurance

The study was approved by the WHO Ethics Review Committee (WHO ERC: MCA00315) and the respective ethics committees at each study site. Written consent in the local language was obtained from the parent/caregiver and in the presence of a witness if the parent/caregiver was illiterate. Oversight was provided by a technical steering committee and a data and safety monitoring board. The trial was performed following the principles of the Declaration of Helsinki.

All CLHWs, supervisors and outcome assessors were trained in iCCM,^3 31^ through the existing training system of the programme. Additionally, CLHWs in intervention clusters were also trained in pulse oximetry and treatment of young infants with oral amoxicillin. Hands-on refresher training was conducted by the investigators and study coordinators every 3–6 months. Study staff, including supervisors and independent outcome assessors, were also trained in pulse oximetry.

Study investigators held preparatory meetings with the Ministry of Health, district health officers and community leaders at each site. CLHWs in India (Accredited Social Health Activists) who had not routinely treated children with pneumonia were trained and evaluated for their ability to do so and verify their acceptance in the community as treatment providers.^32^

### Study procedures

Detailed study procedures and implementation of iCCM by country are published elsewhere.^33^ Briefly, sick young infants in both the intervention and the control clusters were identified by CLHWs in the community through active case finding during home visits and care-seeking by families to CLHWs. Additionally, sick young infants were referred to CLHWs by community health promotion volunteers in Ethiopia and Bangladesh. A standardised assessment of young infants as per iCCM protocol was undertaken.^3 31^ The respiratory rate was counted manually for 1 min using a timer (UNICEF, Copenhagen, Denmark). Respiratory rates of ≥60 breaths per min were confirmed by a second measurement. If the second respiratory rate measurement was different from the first by ≥±2 breaths per min, a third measurement was taken. Infants were classified as having fast breathing pneumonia if two respiratory rate counts were ≥60 breaths per min. A study supervisor re-examined infants identified by the CLHW as fast breathing pneumonia within half an hour to confirm the respiratory rate and other findings. In cases of disagreement, the supervisor’s measurements were considered as the final.

In the intervention clusters, pulse oximetry was performed using the Rad-5v pulse oximeter (Masimo, Irvine, California, USA) by CLHWs, and study supervisors confirmed their measurements to identify hypoxaemia (SpO_2_ <90%).^30^ In contrast, in the control clusters, only study supervisors conducted pulse oximetry to identify hypoxaemic infants. Young infants 7–59 days old with fast breathing only, without danger signs, SpO_2_ ≥90%, with informed written consent and previously not enrolled in this study were enrolled in the study.

The unit of randomisation was Union in Bangladesh, Health Centre in Ethiopia, Health Subcentre in India and Health Centre in Malawi. We used stratified randomisation based on the median population of the cluster. Clusters were divided into two strata as the first stratum with median or above population and the second stratum with less than the median population. An equal number of clusters were randomly selected from each stratum. The randomisation lists were prepared off-site by the WHO coordinating office in Geneva not involved with the study (details are published elsewhere).^33^

### Community-level enhanced and standard pneumonia management

In the intervention clusters, enrolled young infants were treated with oral amoxicillin dispersible tablets (Medreich, UK) by the CLHW according to WHO weight bands 125 mg two times per day for <4 kg body weight and 250 mg two times per day for >4 kg body weight.^5^ The first dose was given by the CLHW and demonstrated to the infant’s parent/caregiver, and subsequent doses were administered by the parent/caregiver at home. If the infant vomited within 30 min of administration of medication, the dose was re-administered. Enrolled infants were followed up on days 2, 4 and 7 after enrolment by the treating CLHW to assess for clinical improvement, need for referral, and adherence to therapy.

In the control group, enrolled young infants were immediately referred to a health facility after pre-referral antibiotic dose for further management, and no study-specific regular follow-up by CLHW was stipulated. However, CLHWs were encouraged to follow-up with patients according to their national guidelines.

### Study outcomes

#### Primary outcome

The primary outcome was treatment failure, defined as (a) death at any time up to 14 days of enrolment; or (b) hospitalised for any reason or has any indication of hospitalisation on day 6 of enrolment (defined as the presence of any sign of deterioration—not able to feed at all or stopped feeding well, convulsion, movement only when stimulated or no movement at all, severe chest indrawing, high body temperature (≥38°C), low body temperature (<35.5°C) or SpO2 <90%) on day 6; or (c) persistence of fast breathing on day 6; or (d) development of a serious adverse event of treatment by day 6 such as anaphylactic reaction, severe diarrhoea or generalised severe rash. The primary outcome was ascertained on day 6 (from #b–d) and day 14 (#a) of enrolment by independent outcome assessors (clinical officers/nurses trained in iCCM and trial methodology) who were unaware of the treatment received by the child to reduce potential measurement bias.

#### Secondary outcome

There are three components of the secondary outcome. First, the feasibility of using a pulse oximeter by CLHWs; second, the accuracy of pulse oximetry when used by CLHWs against a standardised measurement by a trained supervisor; and last, the impact of pulse oximetry on referral and treatment outcomes. Findings of the second and third component of secondary outcome are presented in the current paper. We will present the findings of the first component separately.

### Statistical analysis

#### Sample size

The sample size was calculated by assuming that a cluster had a population of 5000 and a 3% birth rate. Around 150 births were expected in each cluster annually, providing 300 young infants over the 2-year study period. It was expected that about 5% of young infants would develop fast breathing during the first 2 months of life, yielding 15 cases of fast breathing pneumonia per cluster in 2 years. If there is truly no difference between the standard iCCM and enhanced iCCM (assuming 10% treatment failure in both groups),^18 34^ with a design effect of 1.2 and attrition of 10%, then 990 infants with fast breathing per group were required to be 90% sure that the upper limit of a two-sided 95% CI will exclude a difference in favour of the standard iCCM of >5% (assuming >50% difference in the treatment failure between two groups). We expected 10 cases per cluster over the study period, so 198 clusters (99 per group) were required for random allocation.

#### Data collection and analysis

Standard case report forms were used to collect data. Double data entry was done into a centralised database maintained by the Data Coordinating Centre (DCC) in India. Each site uploaded a cleaned database to the DCC each month for quality checks. A planned interim analysis was conducted and reviewed by the data safety and monitoring board (DSMB) after nearly 75% of enrolment. As the enrolment rate was higher in Asian sites, the DSMB recommended to cease enrolment at Asian sites (Bangladesh and India) and continue enrolment at African sites.

Descriptive statistics were used to calculate frequencies and percentages. Based on a predefined analysis plan to test for non-inferiority in the treatment failure rate between the intervention and control clusters, the difference in risk of treatment failure with a 95% CI was calculated and adjusted for site and cluster. Post hoc prespecified subgroup (region, age category, sex, weight-for-age z score category and respiratory rate category) analyses were performed to examine the difference in risk of treatment failure with 95% CI between intervention and control clusters.

For secondary outcomes, descriptive analyses were performed to evaluate the feasibility, accuracy and impact of pulse oximeter use by CLHWs in intervention clusters. All analyses were performed using Stata V.14.2 (StataCorp, College Station, Texas, USA).

### Patient and public involvement statement

The development of the research question was informed by the large burden of pneumonia-related mortality among children worldwide. Patients were not advisers in this study, nor were they involved in the design, recruitment or conduct of the study. Results of this study will be made publicly available through open-access publication where study participants may access them.

## Results

### Study participants

**Figure 1:**
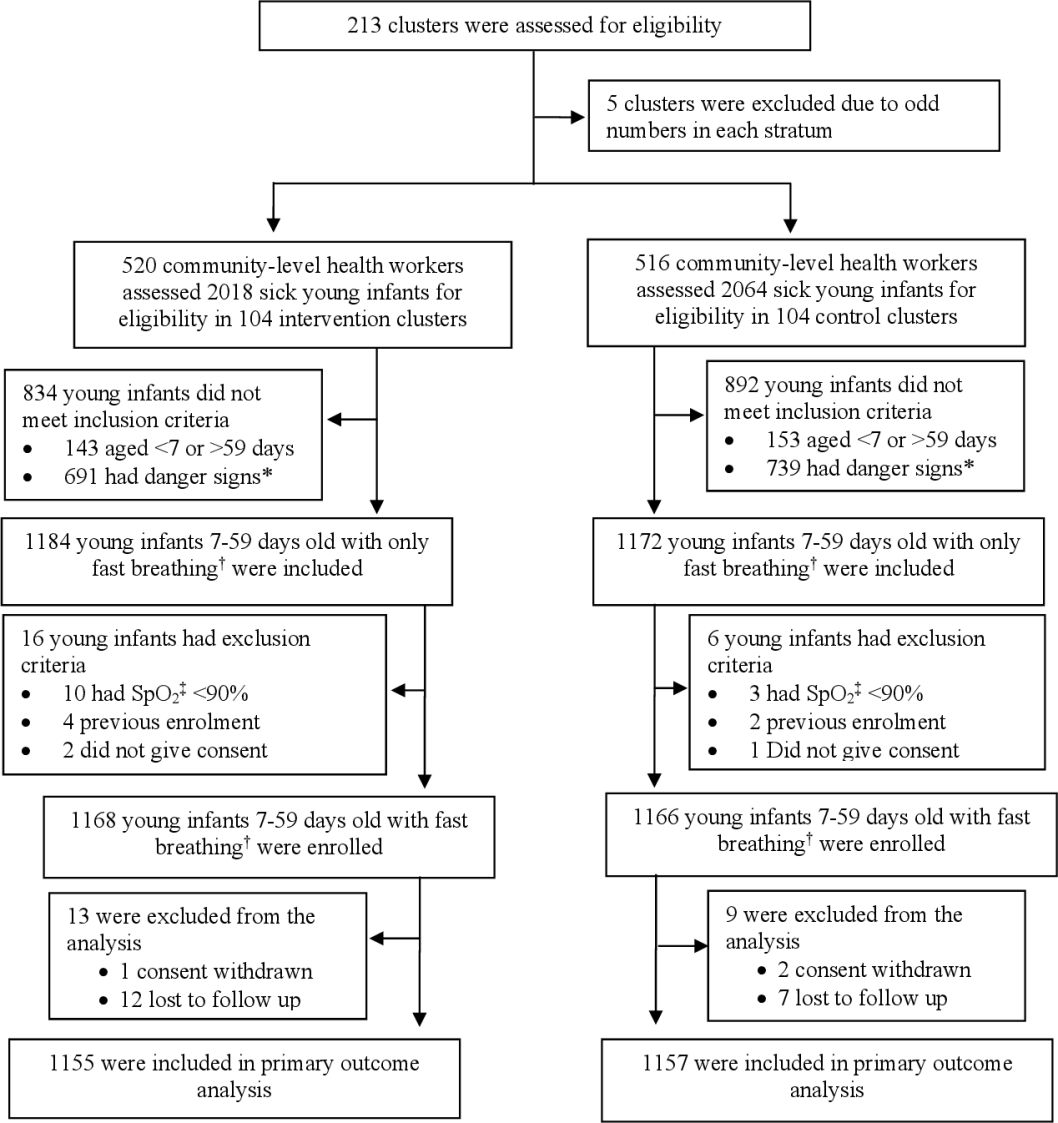
Trial profile. *Danger sign is defined as the presence of any of the following signs: not able to feed at all or stopped feeding well, convulsion, movement only when stimulated or no movement at all, severe chest indrawing, high body temperature (≥38°C), low body temperature (<35.5°C), local infection, yellow soles, diarrhoea, low weight (<2000 g) or SpO_2_ <90%. ^†^Fast breathing is defined as respiratory rate >60 breaths/min. ^‡^SpO2, oxygen saturation.

During the study period, CLHWs assessed 4082 sick young infants (2018 in 104 intervention and 2064 in 104 control clusters) at all four sites (figure 1). Of 2356 infants who had fast breathing only, 13 (0.5%) had hypoxaemia, 6 (0.2%) were previously enrolled and 3 (0.1%) did not give consent. Thus, we enrolled 1168 infants in the intervention and 1166 in the control clusters. We excluded 22 non-hypoxaemic infants with fast breathing from the analysis due to loss to follow-up (n=19) and consent withdrawal (n=3), resulting in 2312 infants (1155 infants in intervention and 1157 in control) for intention-to-treat analysis.

Young infants were enrolled in Bangladesh from 1 December 2016 to 10 May 2018, Ethiopia from 15 September 2016 to 31 December 2018, India from 1 March 2017 to 10 May 2018 and Malawi from 15 December 2016 to 31 July 2017.

**Figure 2:**
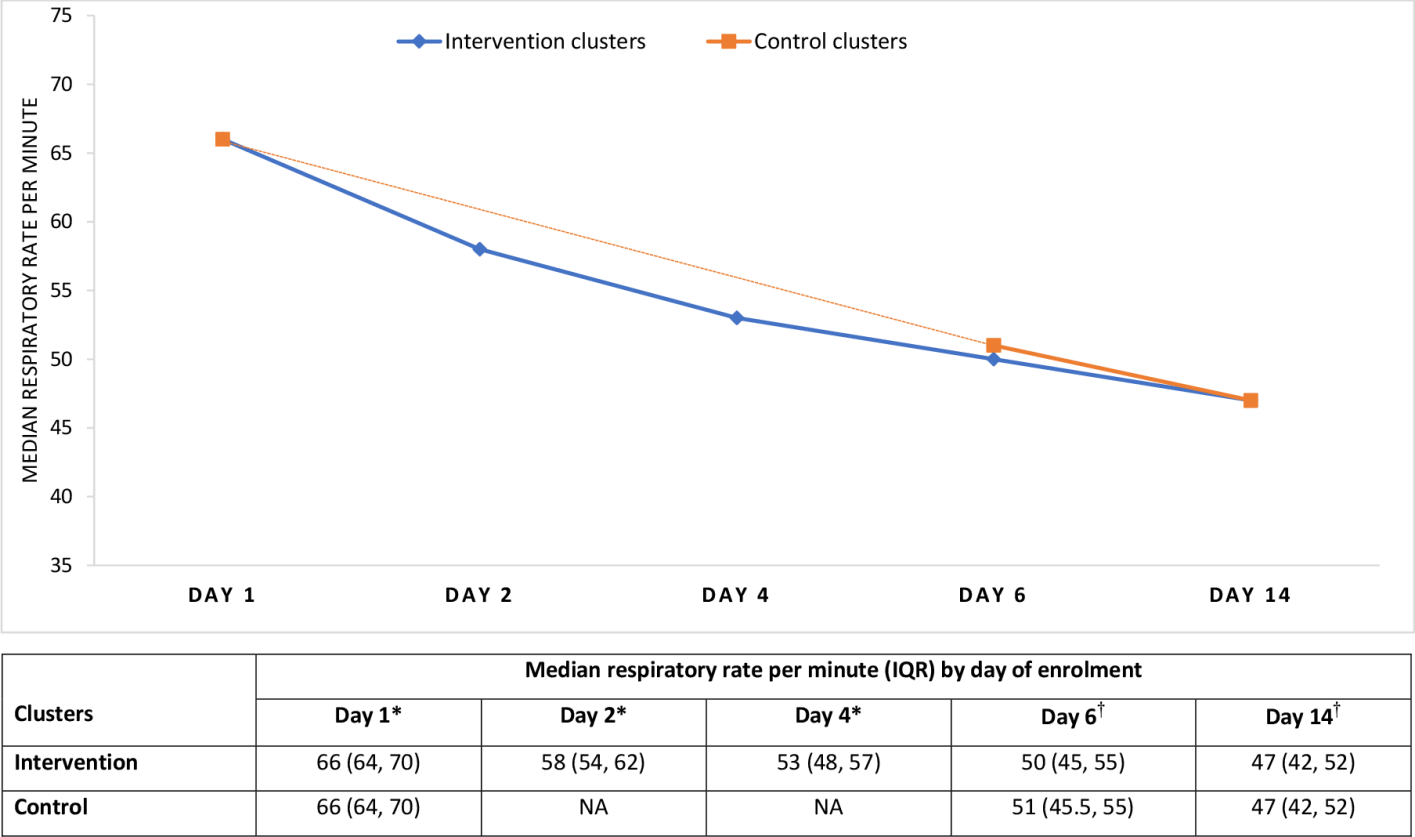
Median respiratory rate per minute by enrolment day of enrolled young infants in intervention and control clusters. *Assessed by community-level health workers. ^†^Assessed by independent outcome assessors.

The cluster and individual level baseline characteristics showed no important differences between the intervention and control groups at all four sites (table 1). The median respiratory rate declined gradually once the treatment was initiated in both groups (figure 2).

**Table 1:** Characteristics of infants at enrolment

	Intervention clusters	Control clusters
**(i) Cluster level characteristics**		
Bangladesh		
Clusters—no.	26	26
Young infants enrolled—no.	211	202
Population size per cluster—median (IQR)	25 000 (21 000–30 000)	25 000 (21 000–28 000)
Community-level health workers per cluster—median (IQR)	13 (11–14)	12 (10–15)
Distance to referral facility per cluster in kilometres—median (IQR)	17 (10–22)	12 (7–20)
Ethiopia		
Clusters—no.	10	10
Young infants enrolled—no.	386	336
Population size per cluster—median (IQR)	24 000 (17 000–31 000)	20 000 (13 000–27 000)
Community-level health workers per cluster—median (IQR)	7 (4–11)	7 (6–10)
Distance to referral facility per cluster in kilometres—median (IQR)	37 (20–47)	22 (10–30)
India		
Clusters—no.	46	46
Young infants enrolled—no.	514	596
Population size per cluster—median (IQR)	10 000 (8000–13 000)	10 000 (8000–13 000)
Community-level health workers per cluster—median (IQR)	9 (8–12)	10 (7–12)
Distance to referral facility per cluster in kilometres—median (IQR)	17 (10–24)	19 (14–27)
Malawi		
Clusters—no.	22	22
Young infants enrolled—no.	57	32
Population size per cluster—median (IQR)	21 000 (17 000–26 000)	21 000 (14 000–30 000)
Community-level health workers per cluster—median (IQR)	6 (2–9)	5 (3–7)
Distance to referral facility per cluster in kilometres—median (IQR)	43 (24–50)	54 (29–60)
**(ii) Patient characteristics**		
Number of infants enrolled at all four sites	(N=1168)	(N=1166)
Age (days)—mean (SD)	30.3 (±15.3)	29.2 (±14.7)
Age 7–28 days—no. (%)	596 (51.1)	609 (52.2)
Age 29–59 days—no. (%)	572 (48.9)	557 (47.8)
Male sex—no. (%)	694 (59.4)	673 (57.7)
Weight-for-age z score*—mean (SD)	−1.14 (±1.09)	−1.14 (±1.15)
Weight-for-age z score ≤2—no. (%)	237 (20.6)	248 (21.5)
Weight-for-age z score ≥2—no. (%)	915 (79.4)	904 (78.5)
Respiratory rate (breaths/min)—mean (SD)	67.7 (±5.7)	67.8 (±6.4)
Respiratory rate between 60–69 breaths/min—no. (%)	842 (72.1)	847 (72.6)
Respiratory rate ≥70 breaths/min—no. (%)	326 (27.9)	319 (27.4)

*Information was missing for 30 infants (16 in intervention clusters and 14 in control clusters), who were excluded from this analysis.

### Primary outcome

By day 14, clinical treatment failure occurred in 63 (5.4%) infants in the intervention and 73 (6.3%) in the control clusters, including two deaths in each. The adjusted risk difference between the intervention and control clusters was −1.0% (95% CI −3.0% to 1.1%). Twelve (1%) infants in the intervention and 19 (1.6%) in the control clusters showed clinical deterioration on day 6 assessment. Serious adverse events of treatment were not observed in either group. The subgroup analyses showed no difference in the risk of treatment failure in the intervention and control clusters (table 2).

**Table 2:** Primary outcome according to treatment groups

	Intervention clusters (N=1155)	Control clusters (N=1157)	Adjusted* risk difference % (95% CI)
**Treatment failure† among all enrolled infants—no. (%)**	63 (5.4)	73 (6.3)	−1.0 (−3.0 to 1.1)
Reasons for treatment failure—no. (%)			
Death at any time up to 14 days of enrolment	2 (0.2)	2 (0.2)	
Hospitalised for any reason or has any indication of hospitalisation‡ on day 6 of enrolment	12 (1.0)	19 (1.6)	
Persistence of fast breathing§ on day 6 assessment	49 (4.2)	52 (4.5)	
Serious adverse event of treatment	0 (0.0)	0 (0.0)	
**Treatment failure† by subgroups**	n/N (%)	n/N (%)	
Region—no./total no. (%)			
African sites	20/436 (4.6)	19/361 (5.3)	−0.9 (−4.4 to 2.5)
Asian sites	43/719 (6.0)	54/796 (6.8)	−1.0 (−3.6 to 1.7)
Age categories—no./total no. (%)			
7–28 days	38/590 (6.4)	42/607 (6.9)	−0.7 (−3.7 to 2.1)
29–59 days	25/565 (4.4)	31/550 (5.6)	−1.4 (−4.2 to 1.4)
Sex—no./total no. (%)			
Male	36/685 (5.3)	43/667 (6.4)	−1.3 (−4.0 to 1.4)
Female	27/470 (5.7)	30/490 (6.1)	−0.4 (−3.3 to 2.5)
Weight-for-age z score¶—no./total no. (%)			
>−2 z score	50/905 (5.5)	54/898 (6.0)	−0.5 (−2.9 to 1.8)
<−2 z score	11/234 (4.7)	17/245 (6.9)	−0.8 (−6.9 to 5.3)
Respiratory rate (breaths per min)—no./total no. (%)			
60–69	35/835 (4.2)	49/841 (5.8)	−1.6% (−3.8 to 0.7)
≥70	28/320 (8.7)	24/316 (7.6)	0.9% (−3.5 to 5.3)

*Adjusted for site and cluster.

†Defined as (a) death at any time up to 14 days of enrolment; or (b) hospitalised for any reason or has any indication of hospitalisation on day 6 of enrolment (defined as the presence of any sign of deterioration—not able to feed at all or stopped feeding well, convulsion, movement only when stimulated or no movement at all, severe chest indrawing, high body temperature (≥38°C), low body temperature (<35.5°C) or SpO2 <90%) on day 6; or (c) persistence of fast breathing on day 6; or (d) development of serious adverse event of treatment by day 6 such as anaphylactic reaction, severe diarrhoea or generalised severe rash. The primary outcome was ascertained on day 6 (from #b–d) and day 14 (#a) of enrolment by independent outcome assessors.

‡Defined as the presence of any danger sign (not able to feed at all or stopped feeding well, convulsion, movement only when stimulated or no movement at all, severe chest indrawing, high body temperature (≥38°C), low body temperature (<35.5°C)) or SpO_2_ <90%.

§Defined as respiratory rate of ≥60 breaths/min.

¶Information was missing in 16 infants in intervention clusters and 14 in control clusters, who were excluded from this analysis. Two infants each failed treatment in intervention and control clusters.

SpO_2_, oxygen saturation.

### Secondary outcomes

CLHWs in intervention clusters were able to perform pulse oximetry in all infants except one with fast breathing (n=1183), and in 94.2% (1050/1115) of the cases performed all steps of pulse oximetry assessment according to the instructions, as per the study supervisors’ assessment (table 3). Pulse oximetry readings by CLHWs were either the same or within 2% point reading for 86.3% (962/1115) of cases compared with the supervisors. Hypoxaemia was observed in 10 (0.8%) infants. All hypoxaemic infants were referred, and all of them survived.

**Table 3:** Use of pulse oximetry by community-level health workers in intervention clusters

Secondary outcome	Young infants with fast breathing*
Feasibility of using pulse oximeter by community-level health workers—no./total no. (%)	
Pulse oximetry performed by community-level health workers (CLHWs)	1183/1184 (99.9)
CLHWs† performed all steps‡ as per instructions	1050/1115 (94.2)
Accuracy of pulse oximetry when used by CLHWs—no./total no. (%)	
Difference in SpO_2_ readings between CLHWs and supervisors	
No	575/1115 (51.6)
1%	248/1115 (22.2)
2%	139/1115 (12.5)
3% or more	153/1115 (13.7)
Impact of pulse oximetry on referral and outcomes—no./total no. (%)	
Hypoxaemic infants§ identified and referred to hospital	10/1183 (0.8)
Alive after 14 days of initiation assessment	10/10 (100.0)

SpO_2_, oxygen saturation.

*Fast breathing is defined as respiratory rate >60 breaths/min.

†Among CLHWs whose pulse oximetry were validated by supervisors.

‡All steps to perform pulse oximetry are: (1) cleaned the equipment before use, (2) turned on the device correctly, (3) selected the correct probe, (4) attached the probe correctly, (5) positioned the infant correctly and (vi) determined the reading correctly.

§Defined as SpO_2_ <90%. CLHWs identified nine hypoxaemic infants, while the supervisor identified one infant.

Compliance with the recommended treatment strategy is shown in online supplemental table 1.

10.1136/bmjgh-2021-006578.supp1 Supplementary data

## Discussion

Our data demonstrate that trained, supervised and equipped with medicines, CLHWs can effectively and safely treat non-hypoxaemic 7–59 days old young infants with fast breathing pneumonia with oral amoxicillin at the community level. Young infants with fast breathing who received community-based treatment from CLHWs had a similar treatment failure rate to those referred to a health facility for facility-based treatment. Thus, community-based treatment for fast breathing pneumonia in young infants by CLHWs was non-inferior to those who received facility-based treatment.

We compared our community-based data with two facility-based trials,^18 34^ in the absence of comparable community-based data, which evaluated the efficacy of oral amoxicillin for the treatment of fast breathing in young infants. In the Pakistani trial, no death was reported, and the treatment failure rate was 2.8% in infants who received oral amoxicillin,^34^ compared with 5.4% treatment failure (including 0.2% deaths) in the intervention arm of our study. In the African trial, 0.5% deaths and 19% treatment failure in the amoxicillin arm were reported.^18^ Our relatively lower rate of deaths compared with the African study could be due to the exclusion of more vulnerable infants such as those 0–6 days old and hypoxaemic infants, and our lower treatment failure rate was probably due to our exclusion of persistence of fast breathing on day 4 of enrolment from treatment failure criteria. The updated WHO IMCI chart booklet 2019 also recommends infants 0–6 days old with fast breathing to be referred to a hospital.^5^

Learnings from our trial experience can inform the country’s iCCM programmes. First, we know that accepting referral advice to health facilities and appropriate care is suboptimal in many low resource settings,^13 14 18^ resulting in preventable pneumonia deaths.^35 36^ Although in our data, the referral advice was accepted by a majority in the control clusters that may be due to the research environment effect. We know that fast breathing pneumonia is common in young infants^6–9 12 18 37 38^; therefore, increasing access to treatment in the community can substantially improve overall health outcomes.^39^ Along with increasing access to treatment, other benefits of treatment observed at the community level are early initiation of treatment and standard case management compared with those referred who may have either received standard treatment or other antibiotics from various sources. Second, the adherence to community-level treatment in the intervention clusters was high due to regular follow-up visits by CLHWs to counsel parents/caregivers. Third, community-level treatment could offer other advantages such as financial savings, ease for the families and higher rates of family satisfaction compared with facility-based care.^40–44^ Fourth, well-trained and supervised CLHWs can classify and treat fast breathing pneumonia well. Fifth, CLHWs can perform pulse oximetry accurately and can identify and refer hypoxaemic young infants to the hospital, thus improving their outcomes. However, the proportion of hypoxaemic young infants with pneumonia at the community level was very small, so given the resource constraints and competing priorities such as preventing amoxicillin stockouts, the use of pulse oximeter at scale at this level needs further study.

Our findings support the community-based treatment of fast breathing pneumonia in young infants by CLHWs, especially in areas with poor access to facility care and humanitarian settings. However, implementation experience is needed at the real-world programme level to learn about barriers and facilitators before it can be fully scaled up. In a programme setting, it is challenging to maintain a high level of training and supervision and household follow-ups on days 2, 4 and 7 of treatment. Thus, the benefits and risks of this approach in a national programme require to be evaluated. Countries need to decide according to their local conditions whether to evaluate the use of pulse oximetry in their programmes at the CLHW level.

Our results also highlight some implementation issues that will be relevant for consideration by large scale iCCM programmes. First, postnatal home visits by CLHWs recommended by the WHO,^45^ and ministries of health that can help identify sick young infants are not routinely performed optimally and would require identifying the barriers and finding solutions to overcome them. Even in well-established iCCM programmes, the practice is not optimal.^46^ Second, sustaining the skills of individual CLHW over time is challenging,^47^ especially in settings where very few sick children are seen every week at the community level. Ongoing supervision and mentoring are required to ensure consistent quality of care.^48–51^ Finally, a regular supply of essential commodities is imperative to avoid stockouts,^51^ which can affect the care-seeking practices.

There are concerns that empiric use of antibiotics for the treatment of pneumonia contributes to antibiotic resistance and that many infections are viral and do not need antibiotics.^52 53^ At the same time, hospitalisation can expose patients to resistant microbes, resulting in serious adverse outcomes, including death.^54^ Data show that the WHO standard case management of pneumonia promotes the rational use of antibiotics in infants and children.^55 56^ Rational drug use through CLHW treatment of pneumonia in Zambia has also been demonstrated.^57^ Young infants with fast breathing with only home care without antibiotics have higher adverse outcomes than those who receive antibiotics.^34^ Until a good point of care test is available to differentiate viral from bacterial infections, empiric antibiotic therapy is a useful tool to reduce pneumonia mortality.

Our study has a few strengths. First, this large multicountry trial leveraged existing iCCM infrastructure across four sites in Africa and Asia and demonstrated that community treatment of young infants with fast breathing pneumonia is safe and feasible across a wide range of settings, seasons and CLHW backgrounds. Prior studies from Ethiopia suggested underutilisation of CLHWs among families of sick young infants,^58 59^ but the presence of health promotion volunteers where CLHWs were not based at the village level (Bangladesh and Ethiopia) resulted in a 44% increase in young infants presenting for care to CLHWs (data not shown). Second, CLHWs used pulse oximetry to identify high-risk hypoxaemic infants and immediately refer them to a hospital for oxygen therapy. Finally, while we used the existing staff to provide iCCM care, the outcomes were determined by an independent outcome assessor not involved in clinical care to reduce potential bias related to subjective assessments.

There are a few limitations of this study. First, the CLHWs in the control clusters did not conduct regular follow-up visits after referral, reflecting the real programme settings compared with the intervention clusters. Second, clinical pneumonia diagnosis was not supported with radiology and microbiology, as this was not feasible in these community settings. A meta-analysis reported the WHO age-related fast breathing thresholds to have a sensitivity of 62% and a specificity of 59% compared with pneumonia diagnosis using chest radiographs.^60^ Third, fast breathing in young infants could be due to viral respiratory infections, high fever, bronchiolitis and malaria, which would not benefit from amoxicillin. Unfortunately, currently, it is difficult to differentiate between viral, bacterial or mixed aetiology in patients with pneumonia when they present, especially at the community level.

## Conclusions

Our trial shows that CLHWs, when trained, supervised and equipped with medicines, can effectively and safely treat non-hypoxaemic young infants 7–59 days old with fast breathing with oral amoxicillin in the community. Translating these findings into practice would increase access to treatment for young infants with pneumonia, especially in settings where access to a health facility is limited. This evidence should be used to review the current WHO/UNICEF iCCM protocol.

## Data Availability

Data are available upon reasonable request.
