# Supplementary material for: Community-based amoxicillin treatment for fast breathing pneumonia in young infants 7–59 days old: a cluster randomised trial in rural Bangladesh, Ethiopia, India and Malawi
Source: BMJ Glob Health. 2021 Aug 20;6(8):e006578. doi: 10.1136/bmjgh-2021-006578 (PMC8381301; doi:10.1136/bmjgh-2021-006578)
Supplement: Supplementary data [file bmjgh-2021-006578supp001.pdf]

Supplementary Table 1: Compliance with recommended treatment strategy in enrolled infants in intervention and control clusters

**Intervention clusters*: 7-day oral amoxicillin treatment (14 doses)** – no. (%)	**Young infants with fast breathing† (N=1153)**
Received treatment for	
Full 7 days (14 doses)	874 (75.8%)
5 to < 7 days (10 to <14 doses)	242 (21.0%)
< 5 days (<10 doses)	37 (3.2%)
**Control clusters‡: Treatment at a health facility following referral** – no. (%)	**Young infants with fast breathing† (N=969)**
Received	
Inpatient treatment in a hospital	85 (8.8%)
Outpatient treatment from any physician clinic/outpatient department of a hospital	800 (82.6%)
Any other treatment	84 (8.6%)

* Missing data of 15 infants were excluded from this analysis.
† Fast breathing is defined as respiratory rate $\geq$ 60 breaths/minute.
‡ Information was not collected from 197 infants enrolled in the first few months of the study, who were excluded from this analysis.
